# Dinitrotoluol (Isomerengemische): MAK-Begründung, Nachtrag

**DOI:** 10.34865/mb2532114xisd9_4ad

**Published:** 2024-12-23

**Authors:** Andrea Hartwig, MAK Commission

**Affiliations:** 1 Institut für Angewandte Biowissenschaften. Abteilung Lebensmittelchemie und Toxikologie. Karlsruher Institut für Technologie (KIT) Adenauerring 20a, Geb. 50.41 76131 Karlsruhe Deutschland; 2 Ständige Senatskommission zur Prüfung gesundheitsschädlicher Arbeitsstoffe. Deutsche Forschungsgemeinschaft, Kennedyallee 40, 53175 Bonn, Deutschland. Weitere Informationen: Ständige Senatskommission zur Prüfung gesundheitsschädlicher Arbeitsstoffe | DFG

**Keywords:** Dinitrotoluol (Isomerengemische), Niere, Kanzerogenität, Keimzellmutagenität, Hautresorption, Genotoxizität, Humanstudien, Epidemiologie

## Abstract

The German Senate Commission for the Investigation of Health Hazards of Chemical Compounds in the Work Area (MAK Commission) summarized and re-evaluated the data for dinitrotoluene (mixtures of isomers) [25321-14-6] considering all toxicological end points, especially for a possible re-classification in Carcinogen Category 1. Relevant studies were identified from a literature search. The critical effect of dinitrotoluene (mixtures of isomers) is nephrocarcinogenicity in rats and mice, which was already described in the last evaluation by the Commission. New studies in a collective of workers in a mining company handling explosives with dinitrotoluene suggest a similar effect in humans. The evidence, however, is limited because neither internal nor external exposure was measured. Instead, inhalation and dermal exposure levels were estimated by the former technical director twenty years after cessation of exposure. Additionally, two different compositions were given for the ingredients of the explosive rods. Therefore, the Commission regards the data as insufficient for a classification in Carcinogen Category 1 and dinitrotoluene (mixtures of isomers) remains classified in Carcinogen Category 2. Since the publication of the last evaluation, new studies investigating the genotoxicity of dinitrotoluene have become available. Dinitrotoluene is clastogenic in the liver of rodents, but not in the bone marrow or peripheral blood. 2,4-Dinitrotoluene demonstrated no clastogenic effect in a dominant lethal assay, but 2,6-dinitrotoluene was not evaluated. Data investigating point mutations in germ cells are, however, not available. Due to this data gap, and because it seems likely that the substance can reach the germ cells, dinitrotoluene (mixtures of isomers) has been classified in Germ Cell Mutagenicity Category 3 B. In a developmental toxicity study in rats, dinitrotoluene (mixtures of isomers) had no adverse effects on the foetus up to 150 mg/kg body weight and day; maternal toxicity (methaemoglobinaemia) occurred at 100 mg/kg body weight and day and above. The designation with “H” is confirmed, as according to the findings of a biomonitoring study in workers and the predictions of skin absorption models, dinitrotoluene penetrates the skin and can increase the carcinogenic risk. Valid data to evaluate the sensitizing potential of dinitrotoluene (mixtures of isomers) are not available for humans or for animals.

**Table:** 

**MAK-Wert **	**–**
**Spitzenbegrenzung **	**–**
	
**Hautresorption (1986)**	**H**
**Sensibilisierende Wirkung**	**–**
**Krebserzeugende Wirkung (1985)**	**Kategorie 2**
**Fruchtschädigende Wirkung **	**– **
**Keimzellmutagene Wirkung (2023)**	**Kategorie 3 B**
	
**BAT-Wert**	**–**
	
	
Synonyma	technisches Dinitrotoluol
Chemische Bezeichnung (IUPAC-Name)	Methyldinitrobenzol
CAS-Nr.	25321-14-6
Formel	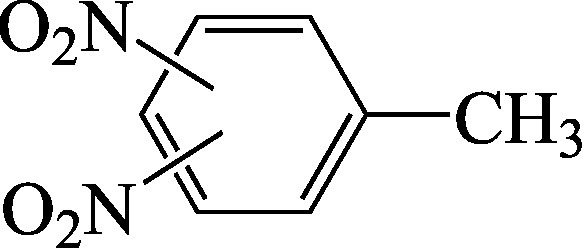
	C_7_H_6_N_2_O_4_
Molmasse	182,14 g/mol
Schmelzpunkt	56–59 °C (ECHA 2020)
Siedepunkt bei 1024 hPa	300 °C mit Zersetzung (Henschler 1986) > 224 °C (ECHA 2020)
Dichte bei 20 °C	1,353 g/cm^3^ (ECHA 2020)
Dampfdruck bei 25 °C	5,3 × 10^–4^ hPa (Henschler 1986) 2,4-Dinitrotoluol: 1,593 × 10^–4^ hPa (ECHA 2020) 2,6-Dinitrotoluol: 3,182 × 10^–4^ hPa (ECHA 2020)
log K_OW_	80/20-Mischung: 2 (ber.) (ECHA 2020) 2,4-Dinitrotoluol: 1,98 (ECHA 2020) 2,6-Dinitrotoluol: 2,1 (ECHA 2020)
Löslichkeit bei 22 °C	166 mg/l Wasser (ECHA 2020) 2,6-Dinitrotoluol: 145 mg/l Wasser (ECHA 2020)
**1 ml/m^3^ (ppm) ≙ 7,558 mg/m^3^**	**1 ml/m^3^ (ppm) ≙ 0,132 ml/m^3^ (ppm)**
	
Hydrolysestabilität	k. A.
Verwendung	zur Herstellung von Toluylendiamin, Toluylendiisocyanat, Farbstoffen, Polyurethanen, Lösungsmitteln, Sprengstoffen, als Gelatinisierungs- oder Imprägnierungsmittel (Wasserdichtigkeit) (ATSDR 2016; IARC 1996; Seidler et al. 2014 a)

Hinweis: Der Stoff kann gleichzeitig als Dampf und Aerosol vorliegen.

Zu dem technischen Gemisch „Dinitrotoluol (Isomerengemische)“ liegt eine Begründung aus dem Jahr 1986 mit der Einstufung in die Kanzerogenitäts-Kategorie 2 (damals Abschnitt III A2) und der Markierung mit „H“ vor (Henschler 1986). 

Das technische Gemisch „Dinitrotoluol (Isomerengemische)“ enthält vor allem 2,4-Dinitrotoluol mit 71–77 % und 2,6-Dinitrotoluol mit 18–20 %. Die weiteren Isomere sind nur in sehr geringer Menge enthalten und machen zusammen maximal 5 % aus, wie 3,4-Dinitrotoluol mit 2,4–4 % und 2,3-Dinitrotoluol mit 1,5–4,3 %. Die bei Raumtemperatur kristallinen gelb oder gelb-orangen Dinitrotoluole sind wasserdampfflüchtig und trotz des geringen Dampfdrucks auch bei Raumtemperatur zu riechen. Beim Erhitzen entstehen toxische Dämpfe von NO_x_ (ATSDR 2016; Henschler 1986).

## 1 Allgemeiner Wirkungscharakter

Die Exposition am Arbeitsplatz kann zu Kopfschmerzen, Müdigkeit, verändertem Geschmack, Appetitverlust, Schwindel, Übelkeit, Schlaflosigkeit und Muskelschwäche führen. Beim Menschen treten in Arbeitsplatzstudien Veränderungen hämatologischer Parameter (Methämoglobinbildung, Anämie, kompensatorische Hämatopoese) und Effekte auf das Nervensystem (klinische Anzeichen von Neurotoxizität , Ataxie, Tremor, schwache Beine, Krämpfe) auf. In Arbeitsplatzuntersuchungen von 1999 bis 2014 wird ein Hinweis auf eine kanzerogene Wirkung im Urogenitaltrakt männlicher Beschäftigter diskutiert. Nieren, Leber, Atemtrakt und Reproduktionssystem sind Zielorgane bei Tieren und es kommt zu Tumoren in den Nieren von Mäusen und in der Leber, den Testes und Brustdrüsen von Ratten.

2,4- und 2,6-Dinitrotoluol sind mutagen im Salmonella-Mutagenitätstest. In Stämmen mit hoher Nitroreduktase- und O-Acetyltransferase-Aktivität sind auch einige Metaboliten mutagen. In Säugerzellen sind die Mutagenitätstests überwiegend negativ, in vivo werden keine Mutationen in Retikulozyten von Ratten nach Exposition gegen 2,6-Dinitrotoluol und keine Mutationen in den Melanozyten von Mäusen nach Exposition gegen 2,4-Dinitrotoluol beobachtet. In der Leber von Nagern werden nach oraler Gabe DNA-Addukte, außerplanmäßige DNA-Synthese, DNA-Schäden (Comet-Assay) sowie Mikronuklei induziert. Im Knochenmark wirken 2,4- und 2,6-Dinitrotoluol sowie 2,4-Dinitrotoluol in den Keimzellen (Dominant-Letaltest) nicht klastogen.

Nach zweijähriger Gabe von 2,4-Dinitrotoluol an männliche Ratten treten ab 0,6 mg/kg KG und Tag neoplastische Noduli und weitere Veränderungen in der Leber auf, nach zweijähriger Gabe von 14 mg 2,4-Dinitrotoluol/kg KG und Tag an CD1-Mäuse Nierendysplasie bei männlichen Tieren.

Für eine hautsensibilisierende Wirkung von 2,3-Dinitrotoluol, 2,4-Dinitrotoluol, 2,5-Dinitrotoluol und 3,4-Dinitrotoluol gibt es keine aussagekräftigen Daten. Für 2,6-Dinitrotoluol gibt es Hinweise auf ein hautsensibilisierendes Potenzial und auf eine photoallergene Wirkung. Zur Atemwegssensibilisierung liegen keine Daten vor.

## 2 Wirkungsmechanismus

Der genaue Mechanismus der kanzerogenen Wirkung ist noch immer nicht abschließend geklärt. Die zu Nitrosoverbindungen und Hydroxylaminen führende Nitroreduktion scheint wie bei der Aktivierung von aromatischen Aminen ein wichtiger erster Schritt bei der Bioaktivierung zu sein. Aus Genotoxizitätsuntersuchungen in vitro und in vivo ist bekannt, dass Dinitrotoluole Genmutationen, DNA-Adduktbildung und Chromosomenaberrationen auslösen und die DNA-Reparatur-Synthese beeinflussen können. Wodurch die sich anschließende promovierende Wirkung, die für die Manifestation der Tumoren notwendig ist, ausgelöst wird, ist jedoch nicht eindeutig belegt (ATSDR 2016; Brüning et al. 2001; Seidler et al. 2014 a).

### 2.1 Nieren

Bei den nach Exposition gegen 2,4-Dinitrotoluol bei Mäusen induzierten Nierentumoren wird ein Schaden am proximalen Tubulus des Nephrons als Auslösereaktion vermutet. 2,6-Dinitrotoluol wirkte in Initiations-Promotions-Untersuchungen an der Leber als komplettes Kanzerogen, 2,4-Dinitrotoluol als Promotor. Als aktiver Metabolit wird das Hydroxylamino-Sulfat des Aminonitrobenzylalkohols gesehen, das instabil ist und zur Bildung von elektrophilen Carbenium- oder Nitreniumionen führen kann, die dann an DNA binden können. 2,6-Dinitrotoluol war negativ in einigen Genotoxizitätstests in vitro und in vivo, in anderen positiv und führte z. B. zu Mikronuklei in der Leber, jedoch nicht im peripheren Blut von oral exponierten Ratten. Auch für den Menschen wird postuliert, dass Dinitrotoluole spezifische Zellen der proximalen Nierentubuli initiieren und es durch eine nachfolgende promovierende Wirkung zu Tumoren im proximalen Nierentubulus kommt. Als mögliche weitere Zielorgane werden aufgrund des Metabolismus der Dinitrotoluole das Urothel des Nierenbeckens und die Harnblase oder allgemein der untere Urogenitaltrakt gese­hen (ATSDR 2016; Brüning et al. 2001; Seidler et al. 2014 a). 

### 2.2 Leber

Für die Entstehung von hepatozellulären Karzinomen bei Ratten, die nach chronischer oraler Gabe von 2,4-, 2,6- und technischem Dinitrotoluol auftraten, wird Toxizität als Ursache gesehen. Erste beobachtete Befunde waren Entzündung, erhöhtes Lebergewicht, hepatozelluläre Hyperplasie und Hypertrophie, Proliferation des Gallengangepithels und ­erhöhte Leberenzyme im Blut. Wahrscheinlich kommt es durch eine sich anschließende promovierende Wirkung zur Tumorentstehung (ATSDR 2016).

## 3 Toxikokinetik und Metabolismus

### 3.1 Aufnahme, Verteilung, Ausscheidung

Wie in der letzten Begründung bereits dargestellt, liegen vor allem Untersuchungen mit dem Hauptbestandteil der Mischung, dem 2,4-Dinitrotoluol vor. Dieses wird bei intraperitonealer oder oraler Gabe an Ratten und Mäuse gut resorbiert mit einer maximalen Blutkonzentration nach sechs Stunden und einer Halbwertszeit von 22 Stunden nach oraler Gabe an Ratten. Die Ausscheidung erfolgt vor allem als Metaboliten mit dem Urin, nur bei einmaligen hohen oralen Dosierungen überwiegend mit den Faeces (Henschler 1986).

Dinitrotoluole werden inhalativ und dermal aufgenommen. Dinitrotoluole und ihre Metaboliten wurden beim Menschen in Urin und Blut nachgewiesen (Seidler et al. 2014 a). In einer arbeitsmedizinischen Studie wurde ein Humanbiomonitoring bei Arbeitern einer Sprengstofffabrik durchgeführt. Aufgrund der niedrigen Dinitrotoluol-Konzentrationen in der Luft und der stark schwankenden und zum Teil hohen Konzentrationen im Urin der Arbeiter wurde darauf geschlossen, dass die dermale Aufnahme der entscheidendere Aufnahmeweg an den Arbeitsplätzen war (Woollen et al. 1985).

Die Halbwertszeit der Metabolitenelimination mit dem Urin betrug bei Beschäftigten in der Dinitrotoluol-Herstellung eine bis 2,7 Stunden (Turner et al. 1985).

Mit den mathematischen Modellen von Fiserova-Bergerova et al. (1990) und IH SkinPerm (Tibaldi et al. 2014) berechnen sich unter der Annahme einer gesättigten wässrigen Lösung (166 mg/l), einem log K_OW_ von 2,0 und einer Molmasse von 182,14 g/mol Fluxe von 8,0 bzw. 0,6 µg/cm^2^ und Stunde. Unter der Annahme einer einstündigen Exposition von 2000 cm^2^ Hautoberfläche würde dies Aufnahmemengen von 16 bzw. 1,2 mg entsprechen.

### 3.2 Metabolismus

**Abb.1:**
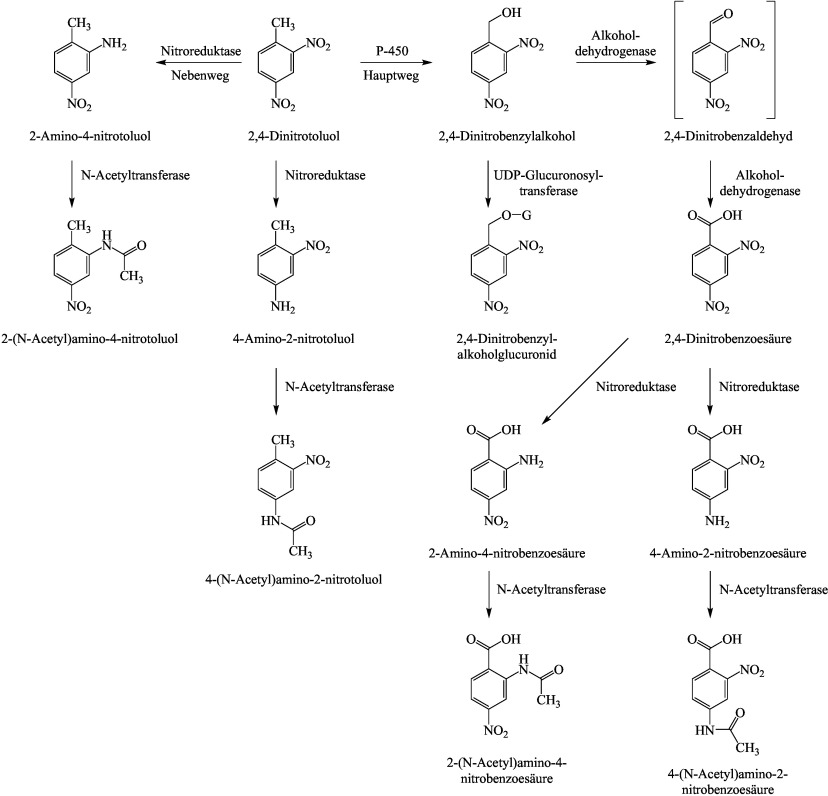
Schema des oxidativen und reduktiven Metabolismus von 2,4-Dinitrololuol nach ATSDR (2016). Postulierte Metaboliten in Klammern

**Abb.2:**
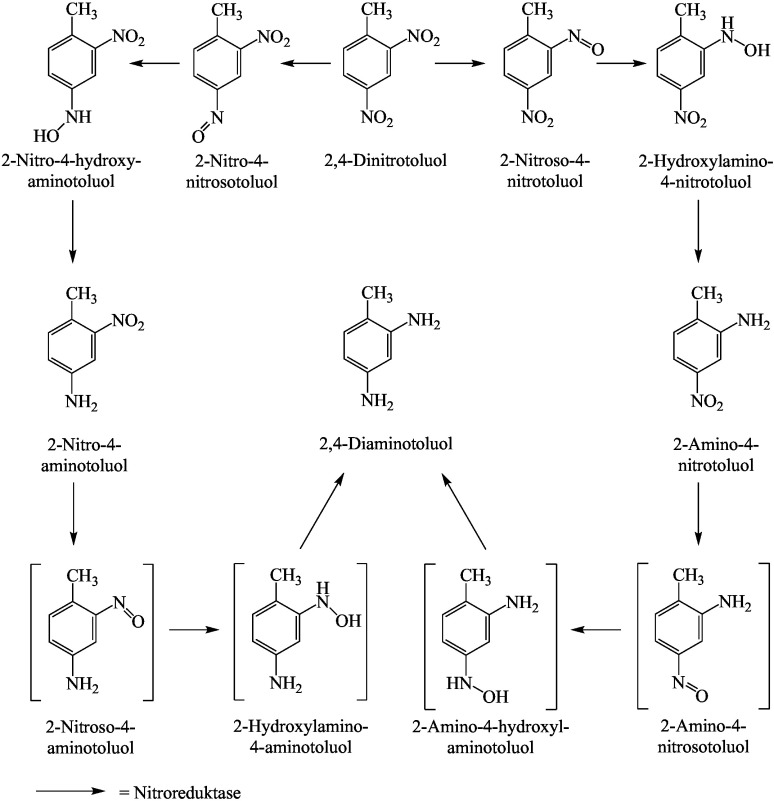
Nitroreduktase-Weg der Metabolisierung von 2,4-Dinitrotoluol nach ATSDR (2016)

**Abb.3:**
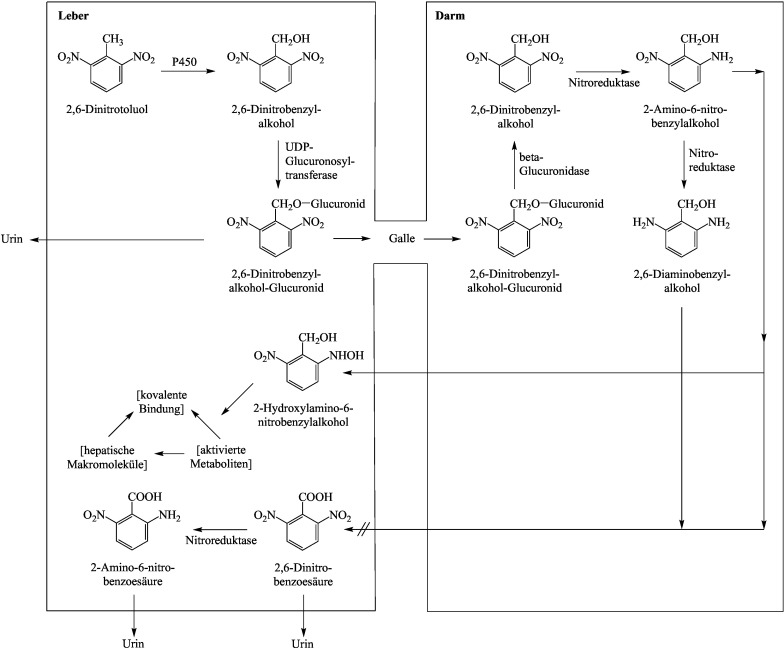
Metabolismusschema von 2,6-Dinitrotoluol nach ATSDR (2016)

Die umfangreiche Datenlage zum Metabolismus der Dinitrotoluole wird anhand der Ausführungen im Bericht der ATSDR (2016) zusammenfassend dargestellt. Die unterschied­lichen Metabolismus-Wege mit den entsprechenden nachgewiesenen Metaboliten bei Ratte und Mensch sind in den Abbildungen 1–3 dargestellt.

#### 3.2.1 Tier

Untersuchungen an Ratten zeigen einen komplexen Metabolismus von 2,4- und 2,6-Dinitrotoluol. Die Metabolisierung erfolgt in der Leber und durch die Mikroflora im Darm.

Oxidativer Weg: 2,4- und 2,6-Dinitrotoluol werden am Cytochrom-P450-Enzymsystem zu den entsprechenden Dinitro­benzylalkoholen oxidiert, die dann entweder glucuronidiert oder weiter über Dinitrobenzaldehyde zu Dinitro­benzoesäuren als Hauptmetaboliten oxidiert werden. Aus diesen können über Nitroreduktasen unterschiedliche Aminonitrobenzoesäuren entstehen. Das Dinitrobenzylglucuronid wird mit dem Urin ausgeschieden oder ­gelangt über die Galle in den Darm, wo es durch die Darmflora über Glucuronidasen und Nitroreduktasen weiter reduktiv metabolisiert wird (siehe reduktiver Metabolismusweg) (ATSDR 2016).

Reduktiver Metabolismus: An Lebermikrosomen von Ratten wurde die Bildung von 2-Amino-4-nitrotoluol und 4-Amino-2-nitrotoluol aus 2,4-Dinitrotoluol über Nitroreduktasen nachgewiesen. Diese Metabolisierung findet aber in deutlich geringerem Umfang in der Leber statt als die oxidative Umsetzung. Ein weiterer reduktiver Metabolismusweg erfolgt über die Darmflora. Alle Isomere wurden von E. coli, die aus dem menschlichen Darm isoliert wurden, über Hydroxylaminonitrotoluole zu Monoaminonitrotoluolen reduziert. Die dekonjugierten Metaboliten werden resorbiert und über den enterohepatischen Kreislauf zurück zur Leber transportiert. In der Leber wird die neu gebildete Aminogruppe durch Cytochrom-P450 N-hydroxyliert und mit Sulfat konjugiert. Das Sulfatkonjugat ist instabil und kann zu einem Carbenium- oder Nitrenium-Ion zersetzt werden, das an hepatische Makromoleküle binden kann. Die Sulfatierung könnte an der Initiationsphase der Hepatokanzerogenese beteiligt sein. Der Stoffwechsel durch die Darmmikroflora scheint für die Produktion von Metaboliten, die kovalent an Makromoleküle der Leber binden, wesentlich zu sein. Es wird auch vermutet, dass reduzierte Metaboliten eine Rolle bei der Entwicklung der Dinitrotoluol-induzierten Methämoglobinämie und Anämie spielen könnten, die bei Menschen und Tieren beobachtet werden (ATSDR 2016).

Bei Ratten wurden Geschlechtsunterschiede im Metabolismus von 2,4-Dinitrotoluol beobachtet. Von männlichen Ratten wird ein größerer Prozentsatz der verabreichten Dosis mit der Galle ausgeschieden als von weiblichen Ratten. Diese scheiden einen größeren Teil der Dosis mit dem Urin als Dinitrobenzylalkohol-Glucuronid aus. Das könnte den Geschlechtsunterschied in der Anfälligkeit der Ratte für die hepatokanzerogene Wirkung von 2,4-Dinitrotoluol erklä­ren: Eine größere Ausscheidung mit dem Urin könnte die Menge des Glucuronids verringern, die der Darmmikroflora für die Umwandlung in einen kanzerogenen Metaboliten zur Verfügung steht (ATSDR 2016).

Die Ergebnisse einer In-vitro-Metabolismus-Studie mit Rattenleber-Homogenat zeigen, dass 2,3- und 2,5-Dinitrotoluol zu Monoaminonitrotoluolen und Hydroxylaminonitrotoluolen reduziert werden (ATSDR 2016). Obwohl es nur sehr wenige Informationen zum Metabolismus von 2,3-, 2,5- oder 3,5-Dinitrotoluol gibt, ist davon auszugehen, dass sie ähnlich wie 2,4- und 2,6-Dinitrotoluol metabolisiert werden.

#### 3.2.2 Mensch

In Arbeitsplatzstudien wurde bei Beschäftigten, die in der Sprengstoffindustrie gegen technische Gemische von Dinitrotoluolen exponiert waren, der Urin auf Metaboliten untersucht. Dabei ist nicht nur die inhalative Exposition zu berücksichtigen, sondern auch die dermale Exposition und auch eine mögliche orale Aufnahme. Der prozentuale Anteil der Hauptmetaboliten 2,4-/2,6-Dinitrobenzoesäure an der gesamten Metaboliten-Ausscheidung mit dem Urin betrug bei Männern ca. 52 % und bei Frauen ca. 29 %. Die inter- und intraindividuelle Schwankungsbreite war sehr groß. Als weitere Metaboliten wurden 2-Amino-4-nitrobenzoesäure (Männer 37 %, Frauen 37 %) und 2,4- und 2,6-Dinitro­­benzylalkoholglucuronid (Männer 9,5 %, Frauen 33,3 %) gemessen. Im Bereich von wenigen Prozenten wurden 4-Amino-­2-nitrobenzoesäure, 2-Amino-6-nitrobenzoesäure, 2-(N-Acetyl)amino-4-nitrobenzoesäure (< 1 %) und 4-(N-Acetyl)­­amino-2-nitrobenzoesäure im Urin nachgewiesen sowie in einigen Proben unmetabolisiertes 2,4- und 2,6-Dinitrotoluol. Bei Beschäftigten in China, die gegen technische Gemische von Dinitrotoluolen und Mononitrotoluolen exponiert waren, war die prozentuale Verteilung der Metaboliten unterschiedlich im Vergleich zu den Kollektiven aus England und Amerika (2,6-Dinitrobenzoesäure 27,3 %, 4-Amino-2-nitrobenzoesäure 26 %, 2-Amino-4-nitrobenzoesäure 21,6 %, 2,6-Dinitrobenzylalkoholglucuronid 18,3 %). Der Metabolismus beim Menschen ist dem der Ratte qualitativ ähnlich. Geringe quantitative Unterschiede in der Bildung der Metaboliten sind wahrscheinlich durch ­andere Aufnahmewege (Mensch inhalativ, Tierversuche meist oral) bedingt. Wie bei Versuchstieren wurde auch beim Menschen ein Geschlechts­unterschied beobachtet: Frauen scheiden einen höheren Anteil der Metaboliten mit dem Urin als Dinitro­benzylalkohol-Glucuronide aus als Männer (ATSDR 2016).

Die Entstehung von Nitrosoverbindungen und Hydroxylaminen durch Nitroreduktion scheint wie bei der Aktivierung von aromatischen Aminen wichtig bei der Bioaktivierung der Dinitrotoluole zu sein (Seidler et al. 2014 a).

## 4 Erfahrungen beim Menschen

### 4.1 Einmalige Exposition

Hierzu liegen keine Daten vor.

### 4.2 Wiederholte Exposition

Die Exposition am Arbeitsplatz kann zu Kopfschmerzen, Müdigkeit, verändertem Geschmack, Appetitverlust, Schwindel, Übelkeit, Schlaflosigkeit und Muskelschwäche führen (Seidler et al. 2014 a).

Zum Zeitpunkt der Begründung aus dem Jahr 1986 (Henschler 1986) lagen vor allem Studien aus den Jahren vor 1950 vor, in denen Arbeiter gegen 2,4-, 2,6- oder das technische Gemisch von Dinitrotoluolen exponiert waren. In diesen Studien fehlen meist Angaben zu Expositionskonzentrationen und Kontrollgruppen, zudem wurden meist kleine Kollektive untersucht. Weiterhin waren die Beschäftigten nicht nur gegen Dinitrotoluole exponiert. Als Zielorgane wurden das Blut (Methämoglobinämie, Anämie, kompensatorische Hämatopoese) und das Nervensystem (klinische neurotoxische Anzeichen wie Kopfschmerzen und Schwindel, Ataxie, Tremor, schwache Beine, Krämpfe) festgestellt. Die Blutparameter reagierten empfindlicher als das Nervensystem. In keiner der Untersuchungen an Arbeitern wurden Wirkungen auf den Atemtrakt berichtet (ATSDR 2016; Henschler 1986).

### 4.3 Wirkung auf Haut und Schleimhäute

Hierzu liegen keine Daten vor.

### 4.4 Allergene Wirkung

#### 4.4.1 Kontaktallergene Wirkung

In einer Publikation wird über drei Personen berichtet, bei denen durch den beruflichen Umgang mit Sprengstoffen ein Verdacht auf eine Allergie gegen Nitroglycerin bestand und in diesem Zusammenhang auch Epikutantests mit 2,4-Dinitrotoluol durchgeführt wurden. 

Ein Mann entwickelte innerhalb eines Jahres einen Juckreiz, welcher sich zu einer Dermatitis an der gesamten Hand weiterentwickelte, nachdem er innerhalb des letzten Jahres vor der Untersuchung etwa zehnmal Umgang mit Dynamit (enthielt 60 % anorganische Nitrate, weitere organische Nitrate und 10 % Dinitrotoluol) hatte. Zuvor hatte der Beschäftigte 14 Jahre als Aufseher von Holzfällern gearbeitet und erst in den letzten vier Jahren begonnen, mit Sprengstoff zu hantieren. Im Epikutantest reagierte der Patient positiv auf 2,4-Dinitrotoluol (2+-Reaktion auf 2-, 1-, 0,5-, 0,2- und 0,1%ige Testzubereitung; fraglich positiv auf 0,05-, 0,02- und 0,01%ige Testzubereitung), sowie auf Dynamit, Nitroglycerin und weitere Nitroverbindungen. Durch das Meiden von Nitroglycerin klangen die Symptome ab. Zwei weitere Personen, welche über viele Jahre in einer Munitionsfabrik bzw. als Sprengberechtigte tätig waren, reagierten negativ auf 2,4-Dinitrotoluol in Konzentrationen zwischen 0,01 und 2 bzw. 1 % (Kanerva et al. 1991). Der Fall erscheint plausibel, jedoch lässt sich aufgrund der Koexposition und weiteren positiven Reaktionen nicht eindeutig nachweisen, dass 2,4-Dinitrotoluol ursächlich für die Dermatitis war. 

#### 4.4.2 Photoallergene Wirkung

Ein Mann, der seit zehn Jahren als Berechtigter für Gesteinssprengung gearbeitet hatte, entwickelte juckende, konfluierende vesikulobullöse Eruptionen an seinen Händen. Die Hautausschläge traten ausschließlich im Sommer auf und nur, wenn seine Hände mit Dynamitpulver in Kontakt kamen. Während des folgenden Winters hatte er keine Beschwerden, im nächsten Sommer traten die Hautausschläge wieder auf, konnten jedoch durch das Tragen von Handschuhen vermieden werden.

Im Photo-Epikutantest (24-stündige okklusive Applikation der Testsubstanzen mit nachfolgender UVA-Belichtung, 5 J/cm^2^, Ablesung 48 Stunden nach Belichtung) kam es zu einer 3+-Reaktion auf Dinitrotoluol (0,5 und 1 % in Erdnussöl), während der unbelichtete Epikutantest negativ blieb. Außerdem reagierte er nur im Photo-Epikutantest mit einer 2+-Reaktion auf Natriumnitrat (5 %), jedoch weder auf die 2,5%ige Testzubereitung noch auf Ammoniumnitrat (2,5 und 5 %). Auf Ethylenglykolnitrat (0,5- und 1%ig in Ethanol) reagierte er sowohl an der bestrahlten, als auch an der unbe­strahlten Hautstelle mit einer 3+-Reaktion. Eine idiopathische Lichtempfindlichkeit wurde ausgeschlossen. Durch das Tragen von Handschuhen kam es zu keinem weiteren Ekzem mehr. Die Autoren folgern eine Photokontaktallergie auf Dinitrotoluol und eine klassische Kontaktallergie u. a. auf Ethylenglykolnitrat. Photo-Epikutantests und Epikutantests mit den Dynamitbestandteilen waren bei fünf bzw. zehn Kontrollpersonen negativ (Emtestam und Forsbeck 1985).

**Tab.1:** Epikutantest- und Photo-Epikutantest-Ergebnisse auf Dynamit-Bestandteile 48 Stunden nach Bestrahlung (Emtestam und Forsbeck 1985)

Testsubstanz	Konzentration	5 J/cm^2^ UVA	Ohne UVA
Ethylenglykolnitrat (in 96 % Ethanol)	1 %	+++	+++
	0,5 %	++	+++
Dinitrotoluol (in Erdnussöl)	1 %	+++	–
	0,5 %	+++	–
Natriumnitrat (in Wasser)	5 %	++	–
	2,5 %	–	–
Ammoniumnitrat (in Wasser)	5 %	–	–
	2,5 %	–	–

### 4.5 Reproduktionstoxizität

Bei chronisch gegen Dinitrotoluol und Toluylendiamin (k. w. A.) in der Luft exponierten Beschäftigten eines Chemiewerkes (21 Exponierte, 9 Kontrollen) war die Spermienzahl statistisch signifikant auf 50 % vermindert und deren Morphologie verändert. Es handelte sich um eine sehr kleine Studiengruppe mit Mischexposition und ohne Angaben zu Konzentration und Expositionsdauer. In vier anderen Studien wurden keine solchen Effekte bei Beschäftigten an ähnlichen Arbeitsplätzen nach Exposition gegen technisches Dinitrotoluol berichtet (ATSDR 2016).

In einer Studie trat ein geringer aber statistisch signifikanter Anstieg an Spontanaborten bei Partnerinnen von chronisch gegen technisches Dinitrotoluol exponierten männlichen Beschäftigten auf. Dieser Befund beruhte jedoch auf sehr wenigen Teilnehmern und es fehlten Informationen zu sonstigen Belastungen der Beschäftigten und deren Partnerinnen (ATSDR 2016). Diese Studie wird daher nicht zur Bewertung herangezogen.

### 4.6 Genotoxizität

Bei 91 Beschäftigten einer Di- und Trinitrotoluol-herstellenden Fabrik in Liaoning, China, zeigte sich im Vergleich zu 60 Beschäftigten der Fabrik, die nicht gegen diese Substanzen exponiert waren, eine statistisch signifikante Erhöhung an Chromosomenaberrationen des Chromatid-Typs einschließlich von Gaps. Die Chromosomenaberrationshäufigkeit (Chromatid-, Chromosom-Typ und Gaps) war statistisch signifikant höher als bei einem weiteren Kollektiv (k. w. A.), das nur gegen Trinitrotoluol exponiert war. Die Beschäftigungsdauer betrug im Median 10,5 Jahre für die Exponierten und 17,6 Jahre für die Kontrollpersonen. Bei den exponierten Beschäftigten war der Anteil an Männern 71 %, bei den Kontrollpersonen 82 %. In der Luft am Arbeitsplatz wurden als 8-Stunden-Mittelwerte Konzentrationen von 759 µg 2-Nitrotoluol/m^3^, 685 µg 4-Nitrotoluol/m^3^, 43 µg 2,4-Dinitrotoluol/m^3^ und 13,9 µg 2,6-Dinitrotoluol/m^3^ gemessen. Als Expositionsmarker wurden unterschiedliche spezifische Hämoglobin-Addukte für 2,4- und 2,6-Dinitrotoluol im Blut gemessen, die nicht mit der Luftkonzentration korrelierten. Die Beschäftigten wurden anhand einer Job-Expositions-Matrix eingruppiert. Das Odds Ratio (OR) für Chromosomenaberrationen bei „hohen“ im Vergleich zu „niedrigen“ Hämoglobin-Adduktspiegeln von 2,4-Dinitrotoluol betrug 1,5 (95-%-KI: 0,75–2,8). Für eine weitere Auswertung erfolgte eine altersabhängige Einteilung in drei Gruppen (jüngste Gruppe 15,9–31 Jahre, Angaben zur Einteilung der anderen Altersgruppen fehlen). Nur in der Gruppe mit den jüngsten Beschäftigten zeigte sich ein statistisch signifikanter Zusammenhang zwischen der Chromosomenaberrationshäufigkeit und der Summe der spezifischen Hämoglobin-Addukte für 2,4-Dinitrotoluol (OR 6,5; 95-%-KI: 1,3–33,3). Die Aussagen der Autoren suggerieren, dass es auch für das Haupthämoglobin-Addukt, gebildet aus 4-Nitroso-2-nitrotoluol, welches entweder oxidativ aus 4-Amino-2-nitrotoluol oder reduktiv aus 2,4-Dinitrotoluol gebildet wurde, einen statistisch signifikanten Zusammenhang gab (Sabbioni et al. 2006). Die Autoren geben keine Erklärung dafür, warum nur bei den jüngsten Beschäftigten ein Zusammenhang bestand. Eine Erklärung könnte sein, dass die mit dem Alter zunehmende Bildung von Chromosomenaberrationen einen möglichen Effekt durch Dinitrotoluol in den anderen Altersgruppen überlagert. Ob die erhöhten Chromosomenaberrationshäufigkeiten ausschließlich dem 2,4-Dinitrotoluol zuzuordnen sind, lässt sich abschließend nicht bewerten, da die Beschäftigten auch gegen andere Nitrotoluole exponiert waren.

### 4.7 Kanzerogenität

Die altersstandardisierte Erkrankungsrate für Nierenkrebs (ICD-10 C64) lag in Deutschland im Jahr 2019 bei 6,5 für Frauen und 15,1 für Männer (pro 100 000 Personen; altersstandardisiert nach altem Europastandard) (RKI 2022).

Eine Kohorte von 4989 Arbeitern einer Munitionsfabrik in den USA, die mindestens einen Tag lang eine Tätigkeit ausübten, bei denen sie gegen Dinitrotoluol exponiert sein konnten, wurden mit 7436 nicht exponierten Kontrollpersonen verglichen, die mindestens fünf Monate im Zeitraum vom 1. Januar 1949 bis 21. Januar 1980 im gleichen Unternehmen arbeiteten. Für 97 % der Arbeiter lagen Gesundheitsdaten ab dem 31. Dezember 1982 vor. Das ­standardisierte Mortalitätsverhältnis (SMR) für alle malignen Erkrankungen betrug 0,84 (95-%-KI: 0,7–1,00). Für Tumoren der Gallen­wege mit sechs beobachteten Fällen ergab sich ein SMR von 2,67 (95-%-KI: 0,98–5,83) im Vergleich zur US-Bevölkerung und ein standardisiertes Ratenverhältnis (SRR) von 3,88 (95-%-KI: 1,04–14,41) im Vergleich zur internen nicht expo­nierten Kontrollgruppe. Das SMR für Tumoren der Gallenwege war für exponierte Arbeiter 10 bis 19 Jahre nach der ersten Exposition am höchsten (SMR: 4,19; 95-%-KI: 0,87–12,35) und niedriger bei den Arbeitern 20 Jahre nach ihrer ersten Exposition (SMR: 1,75; 95-%-KI: 0,21–6,34). Es zeigte sich für diesen Endpunkt keine eindeutige Expositions-Wirkungs-Beziehung in Bezug auf die Exposition gegen Dinitrotoluole, wobei die Zahl an langfristig exponierten Arbeitern sehr gering war und keine quantitative Bestimmung der Exposition gegen Dinitrotoluole und keine Angabe zur Exposition gegen andere Chemikalien vorlag. Die Autoren sehen jedoch einen Hinweis auf eine kanzerogene Wirkung, wie sie im Tierversuch nachgewiesen worden ist (ATSDR 2016; Stayner et al. 1993). 

Es wurde ein Cluster von drei Urothelkarzinomen bei insgesamt 60 Arbeitern gefunden, die in einer Produktionsstätte von Nitrotoluol-Sprengstoffen arbeiteten und gegen hohe Konzentrationen von Dinitrotoluol exponiert waren. Die Karzinome traten zwischen 1990 und 2002 auf und die Inzidenz war 15,9-mal so hoch wie in der Bevölkerung von Sachsen-Anhalt, wo die Produktionsstätte lag. Der Fall 1 war zusätzlich gegen Ammoniumnitrat, Buchenholz, Nitrobenzol, Nitroglykol, Nitrate und Öl exponiert, Fall 2 gegen Nitroglykol, Nitrate, Salpetersäure, Schwefelsäure und Fall 3 gegen Buchenholz, Nitrobenzol und Nitroglykol (Harth et al. 2005).

Aufgrund von Archivunterlagen konnten 500 Arbeiter in untertägigen Kupferminen in Mansfeld in Ostdeutschland als potentiell im Zeitraum 1940 bis 1980 gegen technisches Dinitrotoluol in Sprengstoffen exponiert identifiziert werden. Von diesen konnten 340 kontaktiert und davon 183 in die Studie eingeschlossen werden. Alle Studienteilnehmer waren Deutsche und wurden zu ihrer Exposition befragt. Sie wurden in vier qualitative Expositionsgruppen eingeteilt. Der in der DDR seit den frühen 1950er Jahren ausschließlich verwendete Sprengstoff Donarit^®^ enthielt 30 % technisches Dinitrotoluol, wovon 75 % 2,4- und 20 % 2,6-Dinitrotoluol waren (Brüning et al. 1999). Es liegt über den gesamten Zeitraum der Exposition der Arbeiter kein Messwert zur Expositionskonzentration vor. Über die restlichen 70 % der Zusammensetzung der Sprengstoffstäbe fehlen Angaben in der Veröffentlichung. Donarit-Sprengstoffe können unterschiedliche Zusammensetzungen haben, z. B. gelatinöse industrielle Sprengstoffe: 50 % Ammoniumnitrat, 30 % Dinitrochlorhydrin mit Nitroglykol und 20 % weitere Zuschlagstoffe; oder Ammonsalpeter-Sprengstoffe: 55–84 % Ammoniumnitrat mit bis zu 22 % Nitroglykol und 11–16 % einer Mischung aus Di- und Trinitrotoluol (Haas und Thieme 2023). 

In einer persönlichen Mitteilung wurde der Kommission berichtet, dass der ehemalige technische Leiter der Kupfermine in Mansfeld Gelamon 22 als den damals verwendeten Sprengstoff angibt (Seidler 2023), das nach Literaturangaben (Roschlau et al. 1983) aus 7 % Dinitrotoluol, 22 % Nitroglykol, 20 % Kalk-Ammonsalpeter, 48 % Ammonsalpeter, 0,8 % Nitrozellulose und 2,1 % Holzmehl besteht. 

Die unterschiedlichen Angaben zu den Sprengstoffen, die zum Zeitpunkt der Exposition eingesetzt worden sind, erschwe­ren die Beurteilung. Möglicherweise wurden beide Sprengstoffe verwendet.

Die Beschäftigten waren gegen den Dampf von Dinitrotoluol nach der Sprengung und direkt durch Hautkontakt bei dem Zerbrechen der 70 cm langen Sprengstoffstäbe in kleine Teile und deren Anbringung im Stollen exponiert (keine Verwendung von Handschuhen, 60 % der Arbeiter mindestens fünf Stunden pro Tag damit beschäftigt). Im Zeitraum von 1984 bis 1997 traten sechs Fälle von Urothelzellkarzinomen und 14 Fälle von Nierenzellkarzinomen auf. Die Zeitspanne der Exposition betrug zwischen 7 und 37 Jahre und die Latenzzeit zwischen 21 und 46 Jahre. Im Vergleich zur Allgemeinbevölkerung ergab sich anhand des Krebsregisters von Sachsen-Anhalt eine auf Faktor 4,5 bzw. 14,3 erhöhte Häufigkeit für Nierenzell- und Urothelzellkarzinome. Die 14 Fälle von Nierenzellkarzinomen zeigten keine Expositionsabhängigkeit in Bezug auf die aus einer Job-Expositions-Matrix erwartete Dinitrotoluol-Konzentration im Vergleich zu einer repräsentativen Gruppe nicht exponierter Angestellter. Die Urothelzellkarzinome traten hingegen nur in den Gruppen der hoch exponierten Arbeiter auf. Alle erkrankten Beschäftigten wurden phänotypisiert bezüglich ihres genetischen Status der polymorphen metabolisierenden Enzyme, einschließlich der N-Acetyltransferase 2 und der Glutathion-S-Transferasen M1 und T1. Die Personen mit Urothelzellkarzinomen waren alle langsame Acetylierer. Für die anderen Enzyme zeigte sich kein Zusammenhang zwischen Enzym-Status und Tumor-Häufigkeit (Brüning et al. 1999).

Hoch gegen Dinitrotoluole exponierte Arbeiter (n = 161) der oben beschriebenen Gruppe (Brüning et al. 1999) wurden auf subklinische Nierenschäden untersucht. Die semiquantitative Einteilung in die Expositionsgruppen „niedrig“, „mittel“, „hoch“ und „sehr hoch“ erfolgte anhand von Fragebögen, Expositionsmessungen lagen keine vor. Die Ausscheidung der Biomarker alpha1-Mikroglobulin und Glutathion-S-Transferase alpha mit dem Urin waren expo­sitionsabhängig erhöht. Dies zeigt eine Zunahme an Schäden im proximalen renalen Tubulus (Brüning et al. 2001). 

Diese Daten passen zu dem postulierten Mechanismus der Kanzerogenese: die Dinitrotoluol-Isomere initiieren spezifische Zellen der proximalen Nierentubuli und durch eine nachfolgende promovierende Wirkung kommt es zu Tumoren im proximalen Nierentubulus. Mögliche weitere Zielorgane sind aufgrund des Metabolismus der Dinitrotoluole das Urothel des Nierenbeckens und der untere Urogenitaltrakt (Brüning et al. 2001; Seidler et al. 2014 a).

Aufgrund der Ergebnisse von Brüning et al. (1999, 2001) wurde die Krebsinzidenz in einer Fall-Kohortenstudie bei den männlichen Beschäftigten (Kohorte insgesamt n = 16 441, Teilkohorte n = 999), die im Zeitraum von 1953 bis 1990 in einer von zwei Untertage-Kupferminen in Mansfeld, Ostdeutschland tätig waren, untersucht („externe Analyse“). Da es sich um Arbeiter derselben Minen handelt, die bereits oben beschrieben sind (Brüning et al. 1999), ist von einer Überlappung der Kohorten auszugehen. Bei der Handhabung der Sprengstoffstäbe kommt es zur inhalativen, aber auch zur dermalen Aufnahme, z. B. beim Zerbrechen der langen Sprengstoffstäbe in kleinere Teile. Handschuhe wurden dabei größtenteils nicht getragen. Es liegen keine Messwerte zur inhalativen oder dermalen Expositionskonzentration vor. Die Expositionsbeurteilung erfolgte ca. 20 Jahre nach beendeter Exposition anhand einer Job-Expositions-Matrix eines Experten, des ehemaligen technischen Leiters. Die durchschnittliche Beschäftigungsdauer betrug 6,8 Jahre (0,2–46 Jahre). Angaben zur Krebsinzidenz waren ab dem Jahr 1961 verfügbar und die Nachbeobachtung wurde bis zum Jahr 2005 durchgeführt. Im Zeitraum von 1961 bis 2005 wurden 1476 Krebsfälle beobachtet. Das Krebsrisiko im Vergleich zur Allgemeinbevölkerung in Sachsen-Anhalt wurde mittels standardisierter Inzidenzverhältnisse (SIR) berechnet. Um eine mögliche Verzerrung aufgrund von unterschiedlichen Verlusten in der Nachbeobachtungszeit kontrollieren zu können, wurde eine Sensitivitätsanalyse mit einzelnen Extrapolationsfaktoren für jede 5-Jahre-Geburtskohorte verwendet, anstatt eines globalen Extrapolationsfaktors. Das SIR für alle Krebsarten zusammen war nicht statistisch signifikant erhöht (SIR 1,04; 95-%-Konfidenzintervall (KI): 0,96–1,14), auch die für Nierentumoren (SIR 1,01; 95-%-KI: 0,79–1,27) und Blasentumoren (SIR 1,04; 95-%-KI: 0,82–1,30) nicht. Eine statistisch signifikant erhöhte Krebsinzidenz zeigte sich für Lungentumoren (SIR 1,29; 95-%-KI: 1,13–1,46) sowie ein erhöhtes, jedoch nicht statistisch signifikantes Risiko für Tumoren der Ohrspeicheldrüse und anderen Speicheldrüsen (SIR 1,73; 95-%-KI: 0,72–4,16), letzterer Wert basiert jedoch auf nur fünf Fällen. Bei einer Beschäftigungsdauer von über 20 Jahren zeigte sich ein erhöhtes SIR von 1,41 (95-%-KI: 0,85–2,35) für Blasenkrebs. Für Nierenkrebs waren die SIR ab einer Beschäftigungsdauer von zehn Jahren (SIR 1,51; 95-%-KI: 0,9–2,53) und von mehr als 20 Jahren (SIR 1,25; 95-%-KI: 0,72–2,18) nicht statistisch signifikant erhöht (Seidler et al. 2014 b).

In der Hauptanalyse als Fall-Kohortenstudie („interne Analyse“; Seidler et al. 2014 b) fand die Expositionshöhe der Beschäftigten Berücksichtigung. Es wurden zusätzliche Fälle an Nieren- und Urothelkarzinomen, die im Arbeits­platzregister nicht enthalten waren, durch Daten eines Netzwerkes pathologischer Institute erfasst und in die Auswertung einbezogen. Insgesamt wurden 109 Fälle an Nierenkrebs ausgewertet, von 88 Fällen war die exakte morphologische oder topographische Diagnose verfügbar. Die 109 Fälle und die 993 Nicht-Erkrankten wurden in „nie exponiert“ und „jemals exponiert“ aufgeteilt, wobei die Gruppe der „jemals Exponierten“ nochmals unterteilt wurde nach inhalativer und dermaler, sowie nach niedriger, mittlerer und hoher kumulativer Exposition. In der mittleren und hohen dermalen Expositionsgruppe wurden statistisch grenzwertig bzw. statistisch nicht signifikante erhöhte relative Risiken (RR) für Nierenkrebs beobachtet (mittlere: RR 2,73; 95-%-KI: 1,00–7,42; hohe: RR 1,81; 95-%-KI: 0,75–4,33), ebenso in der Gruppe mit der höchsten inhalativen Exposition (RR 1,36; 95-%-KI: 0,84–2,21). Anzumerken ist, dass die Fallzahlen in den Gruppen mit mittlerer und hoher dermaler Exposition mit 6 bzw. 8 klein waren. Bei der gemeinsamen Auswertung von inhalativer und dermaler Exposition war das RR für Nierenkrebs mit 2,12 (95-%-KI: 1,03–4,37) in der Gruppe „mittel/hoch inhalativ und gleichzeitig mittel/hoch dermal“ statistisch signifikant erhöht. Bei Eingrenzung auf Personen mit einer mindestens 20-jährigen Beschäftigungsdauer im Mansfelder Kupferschieferbergbau findet sich ein auf 1,87 (95-%-KI: 0,97–3,61) erhöhtes Hazard Ratio, basierend auf 36 Fällen. Weiterhin findet sich bei Eingrenzung auf Personen mit einer mindestens 20-jährigen Beschäftigungsdauer im Mansfelder Kupferschieferbergbau ein auf 2,77 (95-%-KI: 1,15–6,71) erhöhtes Hazard Ratio bei kombinierter mittlerer/hoher inhalativer und mittlerer/hoher dermaler Dinitrotoluol-Exposition. Die Arbeitshistorie war bei 34 % der Fälle und bei 57 % der Nicht-Fälle „nur für weniger als zehn Jahre bekannt“. Der Raucherstatus wurde bei 36,7 % der Fälle und bei 40,2 % der Nicht-Erkrankten bestimmt. Bei 5 (6,1 %) Fällen mit auswertbaren Dokumenten gibt es Hinweise auf einen möglichen chronischen Alkoholabusus. Da weder das Rauchen noch der Alkoholmissbrauch mit der Exposition korrelierte, wurden beide Variablen nicht in die Analyse mit einbezogen (Seidler et al. 2014 b). In der Studie erfolgte insgesamt eine sehr gute Datengenerierung und Auswertung. Alle Informationen, die erfasst werden konnten, sind gut dokumentiert. Die Exposition erfolgte durch die verwendeten Sprengstoffstäbe, die Dinitrotoluol enthielten. Zur Zusammensetzung liegen zwei Angaben vor (siehe Studienbeschreibung: Brüning et al. 1999). Die unterschiedlichen Angaben zur Zusammensetzung der Sprengstoffstäbe, die zum Zeitpunkt der Exposition eingesetzt worden sind, erschweren die Bewertung. Die Expositionsbeurteilung erfolgte ca. 20 Jahre nach beendeter Exposition anhand einer Job-Expositions-Matrix eines Experten (ehemaliger technischer Leiter), wobei sowohl die inhalative als auch die dermale Exposition berücksichtigt wurden. Es liegen jedoch weder Messdaten zu Luftkonzentrationen noch Biomonitoring-Daten vor, und auch die Höhe der inhalativen oder dermalen Resorption ist unbekannt. Somit ist die tatsächliche Exposition in Bezug auf die Substanz und ihre Expositionshöhe unbekannt.

## 5 Tierexperimentelle Befunde und In-vitro-Untersuchungen

### 5.1 Akute Toxizität

#### 5.1.1 Inhalative Aufnahme

Zum technischen Gemisch liegen keine Daten vor. Die 6-Stunden-LC_50_ von 2,6-Dinitrotoluol beträgt für männliche und weibliche F344-Ratten 430 mg/m^3^ (56,8 ml/m^3^) (ECHA 2020).

#### 5.1.2 Orale Aufnahme

Für Mäuse bzw. Ratten beträgt die orale LD_50_ für 2,3-Dinitrotoluol 1072 und 1122 mg/kg KG, für technisches 2,4-Dinitrotoluol 1250 und 1000 mg/kg KG, für 2,5-Dinitrotoluol 1231 und 707 mg/kg KG, für 2,6-Dinitrotoluol 1000 und 177 mg/kg KG, für 3,4-Dinitrotoluol 1414 und 1072 mg/kg KG und für 3,5-Dinitrotoluol 607 für weibliche Mäuse und 216 mg/kg KG für weibliche Ratten (Henschler 1986).

#### 5.1.3 Dermale Aufnahme

Hierzu liegen keine Daten vor.

### 5.2 Subakute, subchronische und chronische Toxizität

Zu diesem Abschnitt wurden keine neuen bewertungsrelevanten Daten veröffentlicht. Die in Henschler (1986) aufgeführten Studien zur wiederholten Aufnahme werden nicht nochmals ausführlich dargestellt, sondern nur Studien mit niedrigen Wirkkonzentrationen und Zielorganen auf Basis der Zusammenfassung der ATSDR (2016) berichtet.

#### 5.2.1 Inhalative Aufnahme

Hierzu liegen weiterhin keine Daten vor.

#### 5.2.2 Orale Aufnahme

##### 5.2.2.1 Leber

Technisches Dinitrotoluol und die Isomeren 2,4- und 2,6-Dinitrotoluol induzieren in Langzeituntersuchungen hepatozelluläre Karzinome bei Ratten. Effekte an der Leber von F344-Ratten werden mit 2,4-Dinitrotoluol nach zweijähriger Gabe mit dem Futter ab 0,6 mg/kg KG und Tag (hepatozelluläre Veränderungen, neoplastische Noduli) und mit 2,6-Dinitrotoluol nach 13-wöchiger Gabe ab 35 mg/kg KG und Tag (Gallengangshyperplasie, NOAEL 7 mg/kg KG und Tag) beobachtet. Auch bei Hunden trat nach zweijähriger Gabe von 2,4-Dinitrotoluol biliäre Hyperplasie bei 10 mg/kg KG und Tag mit einem NOAEL von 1,5 mg/kg KG und Tag auf (ATSDR 2016).

##### 5.2.2.2 Nieren

Nach zweijähriger Gabe von 14 mg 2,4-Dinitrotoluol/kg KG und Tag an CD1-Mäuse trat Nierendysplasie bei männlichen Tieren auf. Bei Ratten wurden bis 34,5 mg/kg KG und Tag und bei Hunden bis 10 mg/kg KG und Tag nach zweijähriger Gabe keine Niereneffekte beobachtet (ATSDR 2016).

##### 5.2.2.3 Nervensystem

Effekte auf das Nervensystem wurden in Tierversuchen mit den Isomeren 2,4-, 2,6-, 3,4-, 3,5- und technischem Dinitrotoluol nach subchronischer und chronischer Gabe beobachtet. Es traten dosisabhängig Schwäche, Steifigkeit, starre Lähmung der Hinterbeine, schwerfälliger Gang, Zittern, Ataxie und Krämpfe auf. Die niedrigste Dosis mit Effekten war bei Hunden nach oraler Gabe 25 mg 2,4-Dinitrotoluol/kg KG über einen Zeitraum von zwölf Tagen bis 13 Wochen und 20 mg 2,6-Dinitrotoluol/kg KG und Tag über einen Zeitraum von 13 Wochen. Bei Ratten wurde nach 13-wöchiger Gabe ab 93 mg 2,4-Dinitrotoluol/kg KG und Tag eine Demyelinisierung im Kleinhirn und Gehirnstamm beobachtet. In einer 2-Jahre-Studie mit oraler Gabe an Hunde trat bei einem von sechs Tieren bei 1,5 mg 2,4-Dinitrotoluol/kg KG und Tag ein Verlust der Kontrolle der Hinterläufe auf. Ab 10 mg/kg KG und Tag wurde bei mehreren Tieren Vakuolisierung, Hypertrophie, Mitose des Endothels, fokale Gliose im Kleinhirn sowie perivaskuläre Blutung im Kleinhirn und Gehirnstamm beobachtet (ATSDR 2016).

##### 5.2.2.4 Blut

Durch Dinitrotoluole werden vor allem folgende Blut-Parameter dosisabhängig beeinflusst: Bildung von Met-Hb und Heinz-Körpern, erhöhte Retikulozytenzahl, verminderte Erythrozytenzahl, verminderte Hämatokritwerte und Hämoglobinspiegel. Auch kann als kompensatorischer Effekt eine extramedulläre Erythropoese der Milz auftreten. Hämatologische Befunde wurden nach 14-tägiger oraler Gabe an Ratten mit allen sechs Isomeren von Dinitrotoluol beobachtet. Die geringste kurzzeitig gegebene orale Dosis, die noch zu Effekten im Blutbild führte, war 4 mg 2,6-Dinitrotoluol/kg KG, 14-tägig täglich an Hunde verabreicht. Die geringste längerfristige orale Dosis, die zu Veränderungen im Blutbild führte, war 1,5 mg 2,4-Dinitrotoluol/kg KG und Tag, an Hunde über einen Zeitraum von 3–9 oder 24 Monaten verabreicht. Extramedulläre Erythropoese in der Milz trat bei Hunden ab 4 mg 2,6-Dinitrotoluol/kg KG und Tag bei 4- bis 13-wöchiger Applikation auf (ATSDR 2016).

#### 5.2.3 Dermale Aufnahme

Hierzu liegen keine Daten vor.

### 5.3 Wirkung auf Haut und Schleimhäute

#### 5.3.1 Haut

Die verschiedenen Isomeren wirken an der Haut von Kaninchen nach einer modifizierten Draize-Untersuchung nicht oder leicht bis mäßig reizend (ECHA 2020; Henschler 1986).

In älteren Firmenstudien aus den Jahren 1975 und 1978 waren 2,4- und 2,6-Dinitrotoluol leicht reizend an der Haut von Kaninchen (k. w. A.; ATSDR 2016).

Wurde 10%iges 2,4- oder 2,6-Dinitrotoluol in Erdnussöl auf die geschorene Rückenhaut von Kaninchen gemäß des Draize-Tests aufgetragen, wirkte 2,4-Dinitrotoluol „sehr leicht reizend“ und 2,6-Dinitrotoluol zeigte eine „grenzwertige leichte Reizwirkung“ an der Haut (k. w. A.) (Lee et al. 1975).

#### 5.3.2 Auge

Die verschiedenen Isomeren sind am Auge von Kaninchen nicht reizend (ECHA 2020; Henschler 1986).

In älteren Firmenstudien aus den Jahren 1975 und 1978 wirkten 2,4- und 2,6-Dinitrotoluol in unbekannter Konzentration nicht reizend am Auge von Kaninchen. Eine Untersuchung von 1981 berichtete eine leichte Reizwirkung von 2,4-Dinitrotoluol am Auge von Kaninchen (k. w. A.; ATSDR 2016).

Wurde 10%iges 2,4- oder 2,6-Dinitrotoluol in Erdnussöl in je ein Auge eines Kaninchens gegeben, zeigten beide Substanzen „keine primäre Reizwirkung“ (k. w. A.) (Lee et al. 1975).

### 5.4 Allergene Wirkung

#### 5.4.1 Hautsensibilisierende Wirkung

Es liegen Untersuchungen am Meerschweinchen zu fünf Isomeren vor (2,3-Dinitrotoluol, 2,4-Dinitrotoluol, 2,5-Dinitro­toluol, 2,6-Dinitrotoluol und 3,4-Dinitrotoluol). Es fehlen Informationen zu Positiv- und Negativkontrollen sowie zum Stamm der Tiere (Lee et al. 1975). Laut Angaben der Autoren wurde die Untersuchung nach Magnusson und Kligman (1969) durchgeführt, wobei Angaben zur intradermalen Applikation fehlen. Getestet wurden Konzentrationen zwischen 3 und 5 % in Erdnussöl an jeweils zehn Meerschweinchen (k. w. A.). Bei der Behandlung mit 5 % 2,6-Dinitro­toluol reagierten zwei von zehn Tieren. Trotz der Einschränkungen ist das Testergebnis für 2,6-Dinitrotoluol positiv zu bewer­ten. Schwieriger gestaltet sich die Bewertung der negativen Testergebnisse für 2,3-Dinitrotoluol, 2,4-Dinitrotoluol, 2,5-Dinitrotoluol und 3,4-Dinitrotoluol, da hier jeweils nur zehn Tiere getestet wurden. Negative Testergebnisse sollten jedoch laut Prüfrichtlinie mit 20 Tieren pro Substanz untermauert werden.

**Tab.2:** Hautsensibilisierungstests mit Dinitrotoluol-Isomeren am Meerschweinchen (Lee et al. 1975)

Testsubstanz	Reinheit	Konzentration in Erdnussöl	Reaktion	Bewertung der Autoren	Irritative Wirkung der Testzubereitung an der Kaninchenhaut
2,3-DNT	> 99 %	3 %	0/10	negativ	schwach irritativ
2,4-DNT	98 % 2,4 DNT, 2 % 2,6-DNT	4 %	0/10	negativ	schwach irritativ
2,5-DNT	95 % 2,5-DNT, 1 % 2,3-DNT, 4 % 2,6-DNT	4 %	0/10	negativ	Nekrose
2,6-DNT	> 99 %	5 %	2/10	mild	schwach irritativ
3,4-DNT	> 99 %	3 %	0/10	negativ	schwach irritativ

DNT: Dinitrotoluol

Im computergestützten mechanistischen Prädiktionsmodell DEREK^TM^ wurde für Dinitrotoluol (k. A. zum Isomer) ein negatives Ergebnis erhalten (NIOSH 2011).

#### 5.4.2 Atemwegssensibilisierende Wirkung

Laut Selbsteinstufung im REACH-Dossier der Registranten liegen Daten zur atemwegssensibilisierenden Wirkung vor, aufgrund derer keine Einstufung nach GHS vorgenommen wurde. Die entsprechenden Studien sind jedoch im REACH-Dossier nicht aufgeführt (ECHA 2020).

### 5.5 Reproduktionstoxizität

#### 5.5.1 Fertilität

In einer Drei-Generationenstudie erhielten CD-Ratten 0; 0,0015 %; 0,01 % oder 0,07 % 2,4-Dinitrotoluol mit dem Futter verabreicht (männliche Tiere: 0; 0,6; 3,9; 34,5 mg/kg KG und Tag, weibliche Tiere: 0; 0,7; 5,1; 45,3 mg/kg KG und Tag). Bei der höchsten Dosis fehlte die F2-Generation und es paarten sich nur wenige F1-Tiere, sodass sich ein adverser Effekt auf die Fertilität ableiten lässt (Ellis et al. 1979; Henschler 1986). 

Weitere Generationenstudien wurden seit Erscheinen der Begründung von 1986 nicht durchgeführt. Die Effekte auf die Reproduktionsorgane werden zusammenfassend aus dem Bericht der ATSDR (2016) zitiert.

Effekte auf die Reproduktionsorgane zeigten sich in mehreren tierexperimentellen Studien an Ratten, Mäusen und Hunden. Insbesondere 2,4-Dinitrotoluol führte nach oraler Gabe zu erniedrigter Fertilität und histologischen Veränderungen an männlichen und weiblichen Reproduktionsorganen. Gerade das männliche Reproduktionssystem erwies sich als besonders empfindlich, und es wurden verminderte Spermienproduktion sowie Hodenatrophie, Veränderungen der Sertoli-Zellmorphologie und degenerierte Samenkanälchen festgestellt. Für 2,4-Dinitrotoluol lagen die NOAEL bzw. LOAEL für Effekte auf die männlichen Reproduktionsorgane (Hodenatrophie und reduzierte oder fehlende Spermatogenese) aus 13-Wochen-Studien mit oraler Gabe für Ratten bei 9–34 und 35–371 mg/kg KG und Tag und für Mäuse bei 137–295 und 413–1032 mg/kg KG und Tag. Bei männlichen Ratten war das Ausbleiben der Spermatogenese zum Teil irreversibel. Bei weiblichen Tieren wurden Atrophie der Ovarien und nicht-funktionelle Follikel induziert (ATSDR 2016).

In mehreren Dominant-Letaltests an Mäusen und Ratten führten technisches Dinitrotoluol, 2,4- und 3,5-Dinitrotoluol bis zur höchsten Dosis von 250 mg/kg KG und Tag nach oraler oder intraperitonealer Gabe nicht zur Induktion von Dominant-Letalmutationen (siehe Abschnitt 5.6.2; Ellis et al. 1979; Lane et al. 1985; Soares und Lock 1980).

#### 5.5.2 Entwicklungstoxizität

Seit Erscheinen der Begründung von 1986 wurden keine neuen Studien veröffentlicht.

In einer prä- und postnatalen Entwicklungstoxizitätsstudie wurden F344-Ratten vom 7. bis zum 20. Gestationstag oder vom 7. Gestationstag bis zur Geburt gegen 0; 14; 35; 37,5; 75; 100 oder 150 mg technisches Dinitrotoluol/kg KG und Tag (5 bis 12 Tiere/Dosisgruppe, Vehikelkontrollgruppe: 20 Tiere, Vehikel: Maiskeimöl) mit Verabreichung per Schlundsonde exponiert. Der Untersuchungsumfang der pränatalen Untersuchung am 20. Gestationstag war ähnlich der einer Studie nach der späteren OECD-Prüfrichtlinie 414 (aus dem Jahr 1981). Es ergaben sich bis zur höchsten Dosis keine entwicklungstoxischen oder teratogenen Effekte. Maternaltoxizität trat ab 100 mg/kg KG und Tag in Form von Methämoglobinbildung auf. Bei der höchsten Dosis von 150 mg/kg KG und Tag kam es bei 7/13 Tieren zu rauem Fell, Lethargie und Schwäche der hinteren Gliedmaßen. Bei der postnatalen Untersuchung zeigten sich bei den Nachkommen nicht dosisabhängige Wachstumsverzögerungen (Henschler 1986; Research Triangle Institute 1982).

In der bereits erwähnten Drei-Generationenstudie an Ratten wurde bei der höchsten Dosis von 45,3 mg/kg KG und Tag bei den Nachkommen eine verminderte Überlebensfähigkeit und eine verzögerte Öffnung der Augen festgestellt. Die verringerte Überlebensfähigkeit wurde auf eine Vernachlässigung durch die Muttertiere sowie während der Geburt gestorbene Feten zurückgeführt. Externe Anomalien wurden bei den Nachkommen nicht festgestellt (siehe Abschnitt 5.5.1; Ellis et al. 1979; Henschler 1986).

### 5.6 Genotoxizität

#### 5.6.1 In vitro

Es liegt eine Vielzahl an In-vitro-Untersuchungen mit den Isomeren und dem technischen Gemisch vor, die ausführlich im Bericht von ATSDR (2016) dargestellt sind. Daher erfolgt hier nur eine Zusammenfassung der Daten.

Die verschiedenen Dinitrotoluol-Isomere führen in den meisten Untersuchungen zu Mutationen in verschiedenen Salmonella-typhimurium-Stämmen, jedoch nicht zu Mutationen in Säugerzellen (ATSDR 2016). 2,4- und 2,6-Dinitro­toluol und einige ihrer durch Reduktion gebildeten Metaboliten wirken in Salmonella-Stämmen mit hoher Nitro­reduktase- oder zusätzlich hoher O-Acetyltransferase-Aktivität (YG1041 bzw. YG1042) mutagen (Sayama et al. 1998). Die zu Nitrosoverbindungen und Hydroxylaminen führende Nitroreduktion scheint wie bei der Aktivierung von aromatischen Aminen ein wichtiger erster Schritt bei der Bioaktivierung zu sein (Seidler et al. 2014 a). Der Metabolit 2,6-Di­nitrobenzaldehyd zeigte ein direktes mutagenes Potenzial ohne Zugabe eines metabolischen Aktivierungssystems (Sayama et al. 1989). Der Urin von Beschäftigten aus der DNT- und TNT-Produktion war positiv in dem Salmonella-Stamm YG1041 (Sabbioni et al. 2006). Auch der Urin von Ratten, denen einmalig 75 mg 2,6-Dinitrotoluol/kg KG mit der Schlundsonde verabreicht worden war, war direkt mutagen in Salmonella typhimurium TA98 (ATSDR 2016).

Durch 2,4- und 2,6-Dinitrotoluol wurden Chromosomenaberrationen in Lungenfibroblasten des Chinesischen Hamsters oder humanen peripheren Lymphozyten (Huang et al. 1995) sowie DNA-Schäden (Comet-Assay) in der Rattenleber (Suzuki et al. 2011) und DNA-Einzelstrangbrüche in Sertoli-Zellen der Ratte verursacht (Yang et al. 2005).

#### 5.6.2 In vivo

Auch zahlreiche In-vivo-Untersuchungen mit den Isomeren (ATSDR 2016) und dem technischen Gemisch (US EPA 2013) wurden bereits ausführlich beschrieben und bewertet und werden im Fazit dieses Abschnitts zusammengefasst. 

Im Folgenden werden Tests beschrieben, die nicht in den Tabellen von ATSDR (2016) oder US EPA (2013) aufgeführt sind: 

##### 5.6.2.1 Klastogenitätstests

In 13-Wochen- bzw. 24-Monate-Studien an CD-Ratten wurden mit 2,4-Dinitrotoluol keine Chromosomenaberrationen im Knochenmark oder den Nieren induziert (k. w. A.). Die männlichen und weiblichen Tiere erhielten 13 Wochen lang bis zu 266 bzw. 145 mg 2,4-Dinitrotoluol/kg KG und Tag mit dem Futter verabreicht. In der chronischen Fütterungsstudie betrugen die Dosierungen bis zu 34,5 bzw. 45,3 mg/kg KG und Tag (Lee et al. 1985).

Jeweils drei männliche und drei weibliche SD-Ratten pro Gruppe wurden intraperitoneal entweder an drei aufeinanderfolgenden Tagen (1 × 3) oder drei Wochen lang, dreimal pro Woche an aufeinanderfolgenden Tagen (3 × 3) behandelt. Ein Mikronukleustest an Retikulozyten der SD-Ratte mit 0, 25, 50 oder 100 mg 2,6-Dinitrotoluol/kg KG und Tag verlief negativ, in der Leber wurde jedoch ein dosisabhängiger Anstieg an Mikronuklei beobachtet, statistisch signifikant ab der niedrigsten (1 × 3) und ab der mittleren Dosis (3 × 3) (Dertinger et al. 2019).

Die Schlundsondengabe von 0, 30, 40 oder 50 mg 2,6-Dinitrotoluol/kg KG und Tag an 14 aufeinander folgenden Tagen oder von 0, 20, 30 oder 40 mg/kg KG und Tag an 28 aufeinander folgenden Tagen führte bei männlichen Ratten zu einer statistisch signifikanten Induktion von Mikronuklei in den Hepatozyten. In der 40-mg/kg-Gruppe war der Anteil an Hepatozyten mit Mikronuklei nach 28-tägiger Behandlung höher als nach 14-tägiger. Die histopathologische Untersuchung ergab Effekte bereits ab der niedrigsten Dosis, insbesondere diffuse Hypertrophie der Hepatozyten und perilobuläre Proliferation der Ovalzellen. Nekrose wurde ab der niedrigsten Dosis (14 Tage) beobachtet, jedoch nicht nach der 28-tägigen Behandlung. Im Gegensatz dazu kam es zu einem dosisabhängigen Anstieg von Anisokaryose in allen Gruppen nach längerer Behandlung, nach 14-tägiger Gabe jedoch nur in der Hochdosisgruppe. Im Knochenmark verliefen die Tests negativ. Der Anteil polychromatischer Erythrozyten und der mitotische Index in den Hepatozyten war im Vergleich zur Kontrolle unverändert (Imamura et al. 2015).

Die gleiche Untersuchung wurde auch mit 2,4-Dinitrotoluol mit den Dosierungen von 0, 50, 100 oder 200 mg/kg KG und Tag durchgeführt. Drei von sechs Tieren der höchsten Dosisgruppe verendeten oder waren moribund. Sowohl nach der 14- als auch der 28-tägigen Behandlung kam es zu einem statistisch signifikanten Anstieg an mikronukleihaltigen Hepatozyten ab der niedrigsten Dosis von 50 mg/kg KG und Tag. Im Knochenmark hingegen wurden keine Mikronuklei induziert. Der Anteil polychromatischer Erythrozyten war im Vergleich zur Kontrolle unverändert (Maeda et al. 2015).

##### 5.6.2.2 Mutationen

Ein Pig-a-Test an Retikulozyten und Erythrozyten der SD-Ratte mit 0, 25, 50 oder 100 mg 2,6-Dinitrotoluol/kg KG und Tag verlief negativ. Jeweils drei männliche und drei weibliche Tiere pro Gruppe wurden intraperitoneal entweder an drei aufeinanderfolgenden Tagen oder drei Wochen lang, dreimal pro Woche jeweils an aufeinanderfolgenden Tagen behandelt (Dertinger et al. 2019).

Ein Fellfleckentest an Mäusen mit intraperitonealer Gabe von 100 mg 2,4-Dinitrotoluol (technisch)/kg KG verlief nega­tiv (Soares und Lock 1980).

##### 5.6.2.3 Keimzellen

2,4-Dinitrotoluol induzierte Letalmutationen, aber keine reziproken Translokationen in Drosophila melanogaster (k. w. A.) (ATSDR 2016).

Mehrere Dominant-Letaltests mit DBA/2J-Mäusen, die 250 mg 2,4-Dinitrotoluol (rein), 2,4-Dinitrotoluol (technisch) oder 3,5-Dinitrotoluol/kg KG intraperitoneal und oral erhielten, verliefen negativ (Henschler 1986; Soares und Lock 1980).

Für einen weiteren Dominant-Letaltest erhielten ausgewachsene männliche Sprague-Dawley-Ratten fünf Tage lang täglich 0, 60, 180 oder 240 mg 2,4-Dinitrotoluol/kg KG und Tag gelöst in Maiskeimöl mit der Schlundsonde. Bei Positivkontrollen erfolgte eine einmalige Gabe von Triethylenmelamin. 2,4-Dinitrotoluol induzierte keine frühen fetalen Todesfälle, beeinträchtigte jedoch die Fortpflanzungsleistung (Lane et al. 1985).

#### 5.6.3 Fazit

2,4- und 2,6-Dinitrotoluol sind mutagen im Salmonella-Mutagenitätstest. In Stämmen mit hoher Nitroreduktase- und O-Acetyltransferase-Aktivität sind auch einige ihrer durch Reduktion gebildeten Metaboliten mutagen. In Säugerzellen verliefen die Mutagenitätstests jedoch überwiegend negativ. Auch in vivo werden keine Mutationen in Retikulozyten und Erythrozyten von Ratten (Pig-a-Test, 2,6-Dinitrotoluol) oder in den Melanozyten von Mäusen (Fellfleckentest, 2,4-Dinitrotoluol) beobachtet. 

In der Leber von Nagern, Zielorgan für die kanzerogene Wirkung bei Ratten, werden nach oraler Gabe DNA-Addukte, außerplanmäßige DNA-Synthese, DNA-Schäden (Comet-Assay) sowie Mikronuklei induziert. Im Knochenmark (2,4- und 2,6-Dinitrotoluol), dem peripheren Blut (2,3-, 2,4-, 2,5-, 2,6- und 3,5-Dinitrotoluol) und den Keimzellen (Dominant-Letaltest, 2,4-Dinitrotoluol) von Ratten wirken die untersuchten Dinitrotoluole nicht klastogen. 

Ein Dominant-Letaltest mit 2,6-Dinitrotoluol zur Untersuchung der Klastogenität sowie Mutagenitätstests mit 2,4- und 2,6-Dinitrotoluol in den Keimzellen fehlen.

### 5.7 Kanzerogenität

#### 5.7.1 Kurzzeitstudien

2,4- und 2,6-Dinitrotoluol waren positiv im Zell-Transformationstest in embryonalen Zellen des Syrischen Hamsters (ATSDR 2016).

Das 2,4-Dinitotoluol ist an der Leber ein Tumorpromotor, aber kein Initiator. Dagegen sind 2,6-Dinitrotoluol und das technische Gemisch von Dinitrotoluol sowohl promovierend als auch initiierend an der Leber (ATSDR 2016), wobei die initiierende Wirkung des technischen Gemisches sehr gering war und nur zwölf Stunden nach einer partiellen Hepatektomie auftrat (Henschler 1986; OECD 2004).

Eine 2:1-Mischung aus 2,4- und 2,6-Dinitrotoluol zeigte keine initiierende Wirkung in Kurzzeit-Initiations-Promotions-Untersuchungen an der Sencar-Mäusehaut. Auch je drei i.p. Injektionen pro Woche dieser Mischung über einen Zeitraum von acht Wochen und 16 Wochen Nachbeobachtung führten nicht zur Induktion von Lungentumoren bei Stamm-A-Mäusen (Henschler 1986; OECD 2004).

#### 5.7.2 Langzeitstudien

Wie bereits in der Begründung von 1986 (Henschler 1986) dargestellt, führte die Gabe von 2,4-Dinitrotoluol mit dem Futter in einer Kanzerogenitätsstudie bei CD1-Mäusen zu Nierentumoren, wobei die männlichen Tiere empfindlicher waren (ab 13 mg/kg KG und Tag) als die weiblichen (bei 911 mg/kg KG und Tag). Bei CD1-Ratten traten vor allem hepatozelluläre Karzinome bei männlichen Tieren bei 34 mg/kg KG und Tag (6/29) und bei weiblichen Ratten bei 45 mg/kg KG und Tag (18/34), sowie bei dieser Dosis Tumoren im Mammagewebe (33/35) auf (Ellis et al. 1979). 

Auch nach Gabe von 2,6-Dinitrotoluol mit dem Futter wurden ab 7 mg/kg KG und Tag bei F344-Ratten nach zwölf Monaten hepatozelluläre Karzinome beobachtet (ATSDR 2016).

Das technische Gemisch von Dinitrotoluol (ca. 76,4 % 2,4- und 18,8 % 2,6-Dinitrotoluol) führte in einer Fütterungs-Kanzerogenitätsstudie bei F344-Ratten ab 3,5 mg/kg KG und Tag bei männlichen (♂ 10/130; ♀ 1/129) und bei 14 mg/kg KG und Tag bei männlichen und weiblichen Tieren (♂ 101/128; ♀ 52/130) zu hepatozellulären Karzinomen (Henschler 1986).

Bei 35 mg/kg KG und Tag führte das Gemisch (76 % 2,4- und 18 % 2,6-Dinitrotoluol) in einer 12-Monate-Fütterungsstudie an männlichen Ratten zu hepatozellulären Karzinomen bei 47 % der Tiere, die Fütterung des reinen 2,4-Dinitrotoluols zu keinen hepatozellulären Karzinomen und die Fütterung des reinen 2,6-Dinitrotoluols bei einer Dosis von 14 mg/kg KG und Tag zu hepatozellulären Karzinomen bei 100 % der Tiere (Leonard et al. 1987).

**Fazit**: Das technische Gemisch der Dinitrotoluole ist kanzerogen an der Leber der Ratte und an der Niere der Maus (Tabelle 3).

**Tab.3:** Kanzerogenitätsstudien mit technischem Dinitrotoluol und 2,4-Dinitrotoluol

Autor:	ATSDR 2016; Henschler 1986				
Stoff:	Technisches Dinitrotoluol (76,4 % 2,4-Dinitrotoluol, 18,8 % 2,6-Dinitrotoluol)				
Spezies:	**Ratte**, F344/N, je 130 ♂, ♀				
Applikation:	mit dem Futter				
Dosis:	0; 3,5; 14; 35 mg/kg KG und Tag				
Dauer:	26, 52, 78, 104 Wo, täglich (Zwischenuntersuchung: je 10 Tiere pro Geschlecht und Dosis nach 26 u. 52 Wo, je 20 ♂, ♀/Dosis nach 78 Wo); Hochdosis: überlebende Tiere nach 55 Wo, restliche Dosisgruppen nach 104 Wo				
Toxizität:	ab 3,5 mg/kg KG: Lebertoxizität (Noduli, Zysten, Hyperbasophilie)				
		**Dosis [mg/kg KG u. d]**			
		**0**	**3,5 **	**14**	**35 (52 od. 55 Wo)**
**Tumoren und Präneoplasien**					
**12 Monate **					
**Leber:**					
hepatozelluläre Karzinome	♂	k. A.	k. A.	k. A.	32/40 (80 %)
	♀	k. A.	k. A.	k. A.	15/40 (37 %)
neoplastische Noduli	♂	k. A.	k. A.	k. A.	8/40 (20 %)
	♀	k. A.	k. A.	k. A.	9/40 (22,5 %)
hepatozelluläre Cholangiokarzinome	♂	k. A.	k. A.	k. A.	3/40 (7 %)
	♀	k. A.	k. A.	k. A.	15/40 (37 %)
Cholangiokarzinom	♂	k. A.	k. A.	k. A.	5/40 (12 %)
	♀	k. A.	k. A.	k. A.	0/40 (0 %)
**Testes:**					
Tumoren Interstitialzellen	♂	k. A.	k. A.	k. A.	13/40 (32 %)
**24 Monate**					
**Leber:**					
hepatozelluläre Karzinome	♂	1/120 (0,8 %)	10/130 (8 %)*	98/128 (76 %)*	–
	♀	0/120 (0 %)	1/129 (0,8 %)	45/130 (35 %)*	
neoplastische Noduli	♂	8/120 (7 %)	14/130 (11 %)	64/128 (50 %)*	–
	♀	5/120 (4 %)	15/129 (12 %)*	74/130 (57 %)*	
hepatozelluläre Cholangiokarzinome	♂			15/128 (12 %)	–
	♀			0/130 (0 %)	
**Brustdrüse:**					
Fibroadenome	♂	6/91 (6 %)	0/130 (0 %)	22/79 (28 %)*	–
	♀	16/90 (18 %)	0/129 (0 %)	29/91 (32 %)*	
**Testes:**					
Tumoren Interstitialzellen	♂	82/101 (81 %)	1/1 (100 %)	104/108 (96 %)*	–
**Haut:**					
subkutane Fibrome	♂			44/52 (85 %)	–
	♀			11/15 (73 %)	
Fibrosarkome	♂			10/52 (19 %)	–
	♀			4/15 (27 %)	
Autor:	ATSDR 2016; Henschler 1986; Leonard et al. 1987				
Stoff:	76,5 % 2,4- und 18,8 % 2,6-Dinitrotoluol (2,43 % 3,4-Dinitrotoluol, 1,54 % 2,3-Dinitrotoluol, 0,69 % 2,5-Dinitrotoluol, 0,04 % 3,5-Dinitrotoluol)				
Spezies:	**Ratte**, F344/N, je 28 ♂				
Applikation:	mit dem Futter				
Dosis:	0, 35 mg/kg KG und Tag				
Dauer:	52 Wo, täglich				
Toxizität:	KG-Zunahme ↓, Lebertoxizität				
		**Dosis [mg/kg KG u. d]**			
		**0 **		**35 **	
Überlebende	♂	k. A.		k. A.	
**Leber:**					
hepatozelluläre Karzinome	♂	k. A.		9/19 (47 %)	
neoplastische Noduli	♂	k. A.		10/19 (53 %)	
hepatozelluläre Cholangiokarzinome	♂	k. A.		2/19 (10 %)	
Autor:	Ellis et al. 1979				
Stoff:	**2,4-Dinitrotoluol**				
Spezies:	**Ratte**, CD, je 38 ♂, ♀				
Applikation:	mit dem Futter				
Dosis:	0; 0,57/0,71; 3,9/5,1; 34/45 mg/kg KG und Tag für ♂/♀				
Dauer:	104 Wo, täglich				
Toxizität:	ab 3,9/5,1 mg/kg KG: Effekte, 34/45 mg/kg KG: Überleben ↓				
		**Dosis [mg/kg KG u. d]**			
		**0**	**0,57/0,71**	**3,9/5,1**	**34/45**
Überlebende	♂	32/38	35/38	27/38	34/38
	♀	31/38	36/38	34/38	33/38
**Leber:**					
Foci und hepatozelluläre Veränderungen	♂	9/25 (36 %)	10/28 (36 %)	9/19 (47 %)	16/29 (55 %)
	♀	7/23 (30 %)	18/35 (51 %)	19/27 (70 %)*	13/34 (38 %)
hepatozelluläre Noduli	♂	1/25 (4 %)	2/28 (7 %)	1/19 (5 %)	2/29 (7 %)
	♀	0/23 (0 %)	3/35 (9 %)	2/27 (7 %)	6/34 (18 %)*
hepatozelluläre Karzinome	♂	1/25 (4 %)	0/28 (0 %)	1/19 (5 %)	6/29 (21 %)
	♀	0/23 (0 %)	0/35 (0 %)	1/27 (4 %)	18/34 (53 %)*
**Brustdrüse:** Tumor	♂	0/25 (0 %)	0/28 (0 %)	0/19 (0 %)	2/30 (7 %)
	♀	11/23 (48 %)	12/35 (34 %)	17/27 (63 %)	33/35 (94 %)*
**Haut:** subkutan	♂	2/25 (8 %)	4/28 (14 %)	3/19 (16 %)	17/30 (57 %)*
	♀	1/22 (5 %)	3/35 (9 %)	0/27 (0 %)	6/35 (17 %)
*p ≤ 0,05; nachträglich berechnet mit dem einseitigen Fisher-Exact-Test					
Autor:	Ellis et al. 1979				
Stoff:	**2,4-Dinitrotoluol**				
Spezies:	**Maus**, CD1, je 58 ♂, ♀				
Applikation:	mit dem Futter				
Dosis:	0; 13,5; 95; 900 mg/kg KG und Tag				
Dauer:	104 Wochen, täglich				
Toxizität:	ab 13,5 mg/kg KG: KG-Zunahme ↓				
		**Dosis [mg/kg KG u. d]**			
		**0**	**13,5**	**95**	**900**
Überlebende	♂	33/58	33/58	28/58	ab 10. Mo Mortalität > 60 %, bis 18. Mo 100 % Mortalität
	♀	31/58	29/58	31/58	ab 10. Mo Mortalität > 60 %, bis 21. Mo 100 % Mortalität
**Nieren:**					
Tumoren	♂	0/33 (0 %)	5/33 (15 %)	16/28 (57 %)*	3/40 (7 %)
	♀	0/31 (0 %)	0/29 (0 %)	0/31 (0 %)	1/32 (3 %)
toxische Nephropathie	♂	0/33 (0 %)	3/33 (9 %)	3/28 (11 %)	32/40 (80 %)*
	♀	0/31 (0 %)	3/29 (10 %)	2/31 (6 %)	10/32 (31 %)*
**Leber:**					
Leberzelltumoren	♂	7/33 (21 %)	9/33 (27 %)	8/28 (28 %)	5/40 (12 %)
	♀	2/31 (6 %)	1/29 (3 %)	3/31 (10 %)	1/33 (3 %)
Dysplasie	♂	2/33 (6 %)	14/33 (42 %)*	12/28 (43 %)*	40/40 (100 %)*
	♀	5/31 (16 %)	3/29 (10 %)	5/31 (16 %)	29/33 (88 %)*

*p ≤ 0,05; nachträglich berechnet mit dem einseitigen Fisher-Exact-Test

Mo: Monat; Wo: Woche

## 6 Bewertung

Der kritische Effekt der (technischen) Dinitrotoluole ist die kanzerogene Wirkung an der Leber bei der Ratte und an der Niere bei der Maus.

**Krebserzeugende Wirkung. **Die epidemiologischen Studien, insbesondere die Fall-Kohortenstudie (Seidler et al. 2014 a, b), sind aussagekräftig und legen die im Tierversuch beobachtete kanzerogene Wirkung des technischen Gemisches der Dinitrotoluole auch beim Menschen nahe. Jedoch liegt kein einziger Messwert zur äußeren oder inneren Expositionserfassung der im Zeitraum zwischen 1940 und 1990 exponierten Arbeiter vor (Brüning et al. 1999; Seidler et al. 2014 a, b). Die Expositionsbeurteilung erfolgte ca. 20 Jahre nach beendeter Exposition anhand einer Job-Expositions-Matrix eines Experten, des ehemaligen technischen Leiters der Kupfermine in Mansfeld. Zur Zusammensetzung der verwendeten Sprengstoffstäbe liegen unterschiedliche Angaben vor (Abschnitt 4.7). Eine Zuordnung der beobachteten Effekte zu technischem Dinitrotoluol ist daher nicht eindeutig möglich. Auch waren nur für eine geringe Fallzahl (mittel/hoch inhalativ/dermal exponierte Fälle) die erhöhten Risiken statistisch signifikant. Insgesamt unterstützen die Daten die Einstufung der Dinitrotoluole in Kanzerogenitäts-Kategorie 2 (krebserzeugend im Tierversuch mit Relevanz für den Menschen). Für eine Umstufung nach Kategorie 1 wird die Datenlage besonders aufgrund fehlender Messdaten zum Expositionsnachweis als nicht ausreichend angesehen.

**Keimzellmutagene Wirkung. **2,4- und 2,6-Dinitrotoluol sowie einige der getesteten Metaboliten sind mutagen im Salmonella-Mutagenitätstest. In Säugerzellen verliefen die Mutagenitätstests jedoch überwiegend negativ. Auch in vivo werden keine Mutationen in Retikulozyten und Erythrozyten von Ratten (Pig-a-Test, 2,6-Dinitrotoluol) bzw. in den Melanozyten von Mäusen (Fellfleckentest, 2,4-Dinitrotoluol) beobachtet.

Die Dinitrotoluole sind klastogen in der Leber von Nagern, dem Zielorgan der kanzerogenen Wirkung. Im Knochenmark, dem peripheren Blut und in den Keimzellen von Nagern sind die Klastogenitätstests negativ. Der Dominant-Letaltest wurde nur mit dem 2,4-Isomer durchgeführt, für das 2,6-Isomer besteht daher eine Datenlücke. In den Keimzellen ist der Endpunkt Mutagenität nicht getestet worden. Die Erreichbarkeit der Keimzellen erscheint möglich, aber es liegt kein Nachweis der aktiven Form in den Keimzellen vor. Daher erfolgt eine Einstufung der Dinitrotoluole in die Kategorie 3 B für Keimzellmutagene.

Auch das im Metabolismus gebildete 2,4-Toluylendiamin weist eine ähnliche Datenlage auf und ist in die Kategorie 3 B für Keimzellmutagene eingestuft (Hartwig und MAK Commission 2021).

**MAK-Wert und Spitzenbegrenzung. **Die Ableitung eines MAK-Werts ist wegen der genotoxischen Wirkung nicht möglich. Eine Spitzenbegrenzung entfällt.

**Fruchtschädigende Wirkung. **In einer pränatalen Entwicklungstoxizitätsstudie an Ratten mit Schlundsondengabe von technischem Dinitrotoluol vom 7. bis zum 20. Gestationstag ergaben sich bis zur höchsten Dosis von 150 mg/kg KG und Tag keine entwicklungstoxischen oder teratogenen Effekte. Maternaltoxizität trat ab 100 mg/kg KG und Tag in Form von Methämoglobinbildung auf (Henschler 1986; Research Triangle Institute 1982).

Da kein MAK-Wert aufgestellt werden kann, entfällt die Zuordnung zu einer der Schwangerschaftsgruppen.

**Hautresorption. **Dinitrotoluole sind in vitro und in vivo genotoxisch und in die Kanzerogenitäts-Kategorie 2 eingestuft. Systematische In-vivo- und In-vitro-Untersuchungen zur dermalen Aufnahme von Dinitrotoluolen liegen nicht vor.

Aufgrund der Modellrechnungen (Abschnitt 3.1) und den Ergebnissen der Humanbiomonitoringstudie von Woollen et al. (1985) ist von einer Hautpenetration auszugehen. Der Stoff ist ein nachgewiesenes genotoxisches Kanzerogen, für das keine unbedenkliche Belastung abschätzbar ist. Daher muss auch bei geringen perkutan resorbierten Mengen davon ausgegangen werden, dass das kanzerogene Risiko erhöht ist. Dinitrotoluole bleiben daher mit „H” markiert.

**Sensibilisierende Wirkung. **Zur Bewertung der hautsensibilisierenden Wirkung von 2,3-Dinitrotoluol, 2,4-Dinitrotoluol, 2,5-Dinitrotoluol und 3,4-Dinitrotoluol gibt es keine aussagekräftigen Daten. Eine tierexperimentelle Untersuchung weist auf ein hautsensibilisierendes Potenzial von 2,6-Dinitrotoluol hin. Insgesamt erfolgt für das Gemisch der Dinitrotoluole keine Markierung mit „Sh“. 

Es liegt lediglich ein gut dokumentierter Fall zur photosensibilisierenden Wirkung von 2,6-Dinitrotoluol vor, der ange­sichts der häufigen Verwendung in Sprengstoffen für eine Markierung mit „SP“ jedoch nicht ausreichend ist. 

Zur atemwegssensibilisierenden Wirkung der Dinitrotoluole gibt es keine verwertbaren Daten. Es erfolgt keine Markierung mit „Sa“. 
